# Murine CMV Expressing the High Affinity NKG2D Ligand MULT-1: A Model for the Development of Cytomegalovirus-Based Vaccines

**DOI:** 10.3389/fimmu.2018.00991

**Published:** 2018-05-07

**Authors:** Lea Hiršl, Ilija Brizić, Tina Jenuš, Vanda Juranić Lisnić, Johanna Julia Reichel, Slaven Jurković, Astrid Krmpotić, Stipan Jonjić

**Affiliations:** ^1^Center for Proteomics, Faculty of Medicine, University of Rijeka, Rijeka, Croatia; ^2^Department of Histology and Embryology, Faculty of Medicine, University of Rijeka, Rijeka, Croatia; ^3^Medical Physics Department, University Hospital Rijeka, Rijeka, Croatia; ^4^Department of Physics, Faculty of Medicine, University of Rijeka, Rijeka, Croatia

**Keywords:** murine CMV, cytomegalovirus, vaccine, NKG2D, murine UL16 binding protein-like transcript 1, NK cells, CD8^+^ T cells

## Abstract

The development of a vaccine against human cytomegalovirus (CMV) has been a subject of long-term medical interest. The research during recent years identified CMV as an attractive vaccine vector against infectious diseases and tumors. The immune response to CMV persists over a lifetime and its unique feature is the inflationary T cell response to certain viral epitopes. CMV encodes numerous genes involved in immunoevasion, which are non-essential for virus growth *in vitro*. The deletion of those genes results in virus attenuation *in vivo*, which enables us to dramatically manipulate its virulence and the immune response. We have previously shown that the murine CMV (MCMV) expressing RAE-1γ, one of the cellular ligands for the NKG2D receptor, is highly attenuated *in vivo* but retains the ability to induce a strong CD8^+^ T cell response. Here, we demonstrate that recombinant MCMV expressing high affinity NKG2D ligand murine UL16 binding protein-like transcript (MULT-1) (MULT-1MCMV) inserted in the place of its viral inhibitor is dramatically attenuated *in vivo* in a NK cell-dependent manner, both in immunocompetent adult mice and in immunologically immature newborns. MULT-1MCMV was more attenuated than the recombinant virus expressing RAE-1γ. Despite the drastic sensitivity to innate immune control, MULT-1MCMV induced an efficient CD8^+^ T cell response to viral and vectored antigens. By using *in vitro* assay, we showed that similar to RAE-1γMCMV, MULT-1 expressing virus provided strong priming of CD8^+^ T cells. Moreover, MULT-1MCMV was able to induce anti-viral antibodies, which after passing the transplacental barrier protect offspring of immunized mothers from challenge infection. Altogether, this study further supports the concept that CMV expressing NKG2D ligand possesses excellent characteristics to serve as a vaccine or vaccine vector.

## Introduction

Human cytomegalovirus (HCMV) is a member of herpesvirus family with high seroprevalence rate worldwide (1). After acute infection, cytomegaloviruses (CMVs) establish life-long latency from which periodic reactivations can occur. In immunocompetent individuals CMV is usually asymptomatic, whereas infection in immunocompromised or immunologically immature individuals can cause a severe morbidity (2, 3). In healthy individuals, CMV is controlled by the combined effort of innate and adaptive immunity. While NK cell control is critical during the first days of infection, long-term virus control is maintained predominantly by T cells and antibodies (4–6). Despite several decades of intensive work, no HCMV vaccine is still approved (7).

NKG2D is an activating immune receptor expressed on NK cells, activated and antigen-experienced T cells, and a proportion of NKT and γδ T cells (8). When expressed on NK cells, NKG2D acts as a strong activating receptor, while its engagement on T cells provides co-stimulation (9, 10). Ligands for NKG2D are structurally similar to MHC class I molecules and normally expressed at very low levels. However, upon cellular stress caused by infection or cell transformation, their expression increases leading to NKG2D engagement and activation of immune cells (11, 12). In humans, NKG2D ligands include highly polymorphic MHC-I-related proteins MICA and MICB and UL16-binding proteins (ULBPs) (13). In mice, ligands for NKG2D receptor belong to the family of retinoic acid inducible early transcripts 1 (RAE-1), histocompatibility 60 (H60), and murine UL16 binding protein-like transcript (MULT-1). Although binding to the same receptor, NKG2D ligands differ in their regulation and affinity for the receptor. It is still not fully understood which consequence has the engagement of different NKG2D ligands on the functional outcome of NK and T cell response.

The best evidence that NKG2D engagement plays an important role in immunosurveillance of CMV is the fact that CMV possesses several immunoevasion genes, which prevent the surface expression of NKG2D ligands (14, 15). In MCMV, the majority of these genes belong to the m145 family of immunoevasins which includes *m145, m152*, and *m155* gene products targeting MULT-1, RAE-1, and H60, respectively (16–18). In addition, viral FcRγ receptor encoded by the *m138* gene has been shown to downmodulate the expression of H60, MULT-1, and RAE-1ε ligands (19, 20).

Murine CMV mutants lacking proteins involved in the regulation of NKG2D ligands are attenuated *in vivo* by NK cells. We exploited this knowledge of NKG2D immunoevasion to develop novel CMV-based vaccine vectors. Recombinant MCMV expressing NKG2D ligand RAE-1γ, inserted in a place of its viral regulator *m152* is severely attenuated *in vivo*, but nevertheless induces strong antigen-specific CD8^+^ T cell response to CMV and vectored antigens, providing long-term protection against bacterial infection and tumors (21–23). Likewise, HCMV expressing ULBP2 in place of its viral regulator is susceptible to control by NK cells, but preserved the ability to stimulate HCMV-specific T cells (24).

In this work, we constructed new MCMV-based vaccine vector expressing NKG2D ligand MULT-1 in place of its viral regulator *m145*. Based on our previous results on RAE-1γMCMV, we hypothesized that MULT-1MCMV would also be efficiently controlled while retaining ability to induce potent CD8^+^ T cell response. Indeed, MULT-1MCMV was dramatically attenuated *in vivo* by NK cells and virus was cleared more rapidly than RAE-1γMCMV. Nevertheless, MULT-1MCMV induced a strong CD8^+^ T cell response and anti-viral antibodies. This study further supports our previous results showing that recombinant CMVs expressing NKG2D ligands can be utilized as efficient vaccines and vaccine vectors.

## Materials and Methods

### Construction of Recombinant MCMV Viruses

Wild-type (WT) MCMV refers to a bacterial artificial chromosome (BAC)-derived mouse cytomegalovirus, MW97.01, previously shown to be biologically equivalent to the MCMV Smith strain (VR-1399). Construction of WT MCMV, Δ*m152*MCMV, and RAE-1γMCMV expressing SIINFEKL was described previously (21, 22, 25). MULT-1MCMV, MULT-1MCMV expressing SIINFEKL and Δ*m145*MCMV expressing SIINFEKL were constructed according to published procedure (26). Briefly, for construction of MULT-1MCMV an ORF encoding FLAG-tagged MULT-1 was first cloned into a plasmid containing kanamycin resistance gene (KanR), I-SceI restriction site, and HCMV immediate early promoter (hMIEP) upstream of the cloning site (kind gift from Martin Messerle). The MULT-1 expression cassette containing KanR was PCR amplified using primers 5′-GGGTTAAAACCGCACACAGATGTAGGGGCAGACTCTGAGGACCGGTGTTTCAACTCCGCGGTTGACATTGATTATTGACT-3′ and 5′-GTGAGGGGATTATGTCCTGTTTATTGTC-TCACGACAGACATACAGAGATTCGGACAGTCATCATGGGATCCCGTCGATGT-3′, which contained 60 nucleotides at their 5′ ends homologous to the intended integration site in the BAC-cloned MCMV genome, thereby replacing the *m145* ORF following the homologous recombination. To swap the sequence of D^d^ restricted antigenic m164_167–175_ peptide AGPPRYSRI with K^b^ restricted peptide SIINFEKL, linear DNA fragment was generated using KanR as a template and primers 5′-GCCGTTCGGAAAGGACTACTGTCGGACGTGGGGCGCTGACAGTATAATCAACTTTGAAAAACTGAGGATGACGACGATAAGT-3′ and 5′-AAGGTCTCCTCGCCCGCTGCCACGATGG-CCTGGTTGTTGACGGCCCAGAACAGTTTTTCAAAGTTGATTATACTGTCAGCGCCCCACCAACCAATTAACCAATTC-3′ for PCR amplification. Δ*m145*MCMV expressing SIINFEKL was constructed by deletion of MULT-1 from MULT-1MCMV expressing SIINFEKL according to the same procedure using 5′-GGGTTAAAACCGCACACAGATGTAGG-GGCAGACTCTGAGGACCGGTGTTTCAACTCCGCGAGGATGACGACGATAAGTAG-3′ and 5′-GATTATGTCCTGTTTATTGTCTCACGACAGACATACAGAGATTCGGACAGCGCGGAG-TTGAAACACCGGTCCTCAGAGTCTGCCCCTACACAACCAATTAACCAATTCTG-3′ primers resulting in *m145* deletion.

### Cells and Virus Propagation

BALB/c mouse embryonic fibroblasts (MEF) were grown according to published procedure (27). MEF and SVEC4-10 cells were infected with 1.5 or 3 PFU/cell, respectively. Viruses were propagated on MEF and concentrated by sucrose gradient ultracentrifugation (28). To assess virus replication *in vitro* by multi-step growth kinetics assay, MEF were infected with 0.1 PFU/cell of WT MCMV, RAE-1γMCMV, and MULT-1MCMV. Supernatants were harvested at indicated times after infection and virus titers were determined by plaque assay (28).

### Mice and Infection

C57BL/6, congenic C57BL/6 (Ly5.1/CD45.1^+^), NKG2D^−/−^ (29), BALB/c, TCR transgenic mice specific for M38 (Maxi) (30), and SIINFEKL (OT-1) (31) were bred under specific pathogen-free conditions at the Faculty of Medicine, University of Rijeka. All experiments performed in this study were approved by the Animal Welfare Committee of the University of Rijeka. Unless otherwise noted, gender matched mice at age of 8–16 weeks were infected with 2 × 10^5^ PFU of tissue culture derived recombinant MCMV either in the footpad (f.p.) or intravenously (i.v.). Newborn BALB/c mice were infected intraperitoneally (i.p.) with 500 PFU of indicated viruses 6 h after birth. Newborn C57BL/6 mice were infected i.p. with 200 or 500 PFU of indicated viruses 24 h post-partum. *In vivo* blocking of NKG2D and depletion of NK cells was performed by i.p. injection of mouse α-mouse NKG2D blocking antibody (generated by Center for Proteomics, University of Rijeka, Faculty of Medicine, clone NKG2D.03) or mouse α-mouse NK1.1 (clone PK136) (32) and rabbit α-asialo GM1 antiserum (αAGM1) (Wako Chemicals), respectively. Viral titers from organs were determined by a plaque assay (28).

### Adoptive Transfer

For adoptive transfer experiments C57BL/6 or C57BL/6 CD45.1^+^ mice were immunized f.p. with 2 × 10^5^ PFU of indicated viruses. After 6 weeks, total CD8^+^ T cells were enriched from splenocytes using CD8a^+^ T Cell Isolation Kit (Miltenyi) and sorted on BD FACSAriaII. Adult C57BL/6 recipients were administrated i.p. with 250 µg of depleting NK1.1 antibody (PK136) 1 day prior to sublethal irradiation with 7 Gy. Next day, mice were i.v. injected with 10^6^ sorted CD8^+^ T cells. Newborn C57BL/6 mice were i.p. injected with 10^5^ sorted CD8^+^ T cells 1 day prior to infection.

### *In Vitro* Stimulation of CD8^+^ T Cells

Bone marrow cells isolated from both femurs and tibias of C57BL/6 mice were differentiated into bone marrow-derived dendritic cells (BMDCs) in the presence of J558 supernatant for 6 days (33). BMDCs were infected with 2 PFU/cell of indicated viruses. After 24 h of infection corresponding amount of either Maxi or OT-1 splenocytes was added at different T:E (BMDCs:CD8^+^) ratios, together with Brefeldin A (Sigma). After 6 h of co-incubation, intracellular staining for IFN-γ production was performed.

### Flow Cytometry and Immune Assays

For staining of cell surface expression of NKG2D ligands, mouse NKG2D protein fused with human Fc fragment was used, followed by conjugated donkey α-human IgG Fc secondary antibody (Jackson Immunoresearch). Alternatively, mouse α-mouse RAE-1γ (generated by Center for Proteomics, University of Rijeka, Faculty of Medicine, clone RAE-1γ0.01) or rat α-mouse MULT-1 (clone 1D6) (18) was used, followed by fluorochrome conjugated goat α-mouse IgG (BD Pharmingen) and goat α-rat IgG F(ab′)_2_ (Santa Cruz) secondary antibody, respectively. As isotype control irrelevant protein fused with human Fc was used (human PVR-Fc) or antibodies of same isotype originating from the same host, respectively. Splenocytes from immunized C57BL/6 mice were either immediately stained with fluorescently labeled antibodies for assessment of CD8^+^ T cell phenotype or incubated in the presence of 5 μg/ml H-2^b^ restricted custom synthesized peptides of M45 (_985_HGIRNASFI_993_), M38 (_316_SSPPMFRV_323_), and ovalbumin (_257_SIINFEKL_264_) (JPT Peptide Technologies) in presence of Brefeldin A (Sigma) for cytokine production. After 4 h of stimulation, cells were stained for viability, expression of surface markers, and intracellular cytokines. Reagents used in flow cytometry analysis were purchased from eBioscience/Thermo Fischer Scientific and included: Fixable Viability Dye, αCD8 (clone 53-6.7), αCD44 (clone IM7), αCD62L (clone MEL-14), αCD127 (clone SB/199), αIFNy (clone XMG1.2), and αTNF (clone MP6-XT22) antibodies. Following reagents were obtained through NIH Tetramer Core Facility: H-2K(b) MCMV M38 (_316_SSPPMFRV_323_), H-2L(d) MCMV IE1 (_168_YPHFMPTNL_176_), and H-2D(d) MCMV m164 (_257_AGPPRYSRI_265_). SIINFEKL-specific multimer H-2Kb/SIINFEKL MHC multimer was kindly provided by Dirk Busch (Munich). Samples were analyzed on BD FACSAriaII using FACSDiva and FlowJo (Tree Star, Inc.).

### ELISA

For detection of MCMV-specific total IgG in immune sera, high binding ELISA plates were coated with lysate of Δ*m138*MCMV infected MEF as previously described (34). Shortly, sera of mice were incubated overnight at +4°C, followed by extensive washing and detection with peroxidase conjugated goat α-mouse IgG (H + L) (Jackson Immunoresearch).

### Statistical Analysis

Statistical significance was calculated by unpaired two-tailed Student’s *t-*test or Mann–Whitney *U* test for statistical analyses of the virus titers. Differences in MCMV-specific antibody titers in sera of immune animals were analyzed using two-way-ANOVA and Bonferroni *post hoc* test. *P* values less than 0.05 were considered significant. Only statistically significant differences are indicated in figures. All data were analyzed using GraphPad Prism 5 software.

## Results

### Construction and *In Vitro* Characterization of Recombinant MCMV Expressing MULT-1

**Figure 1:**
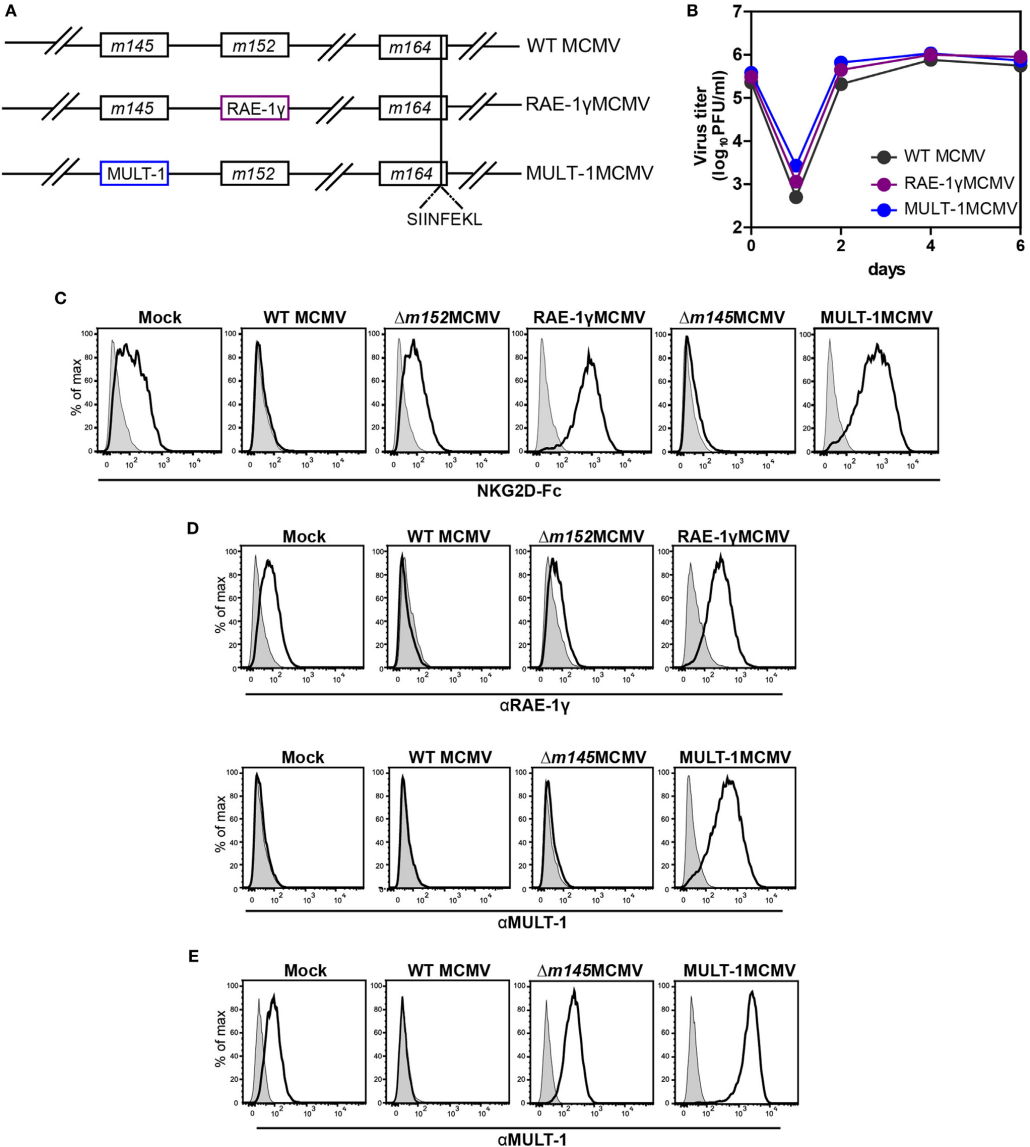
Recombinant viruses used in this study. **(A)** Recombinant murine CMV (MCMV) was made by insertion of genes for NKG2D ligands, RAE-1γ, and murine UL16 binding protein-like transcript-1 (MULT-1) in place of *m152* and *m145*, respectively. OVA-derived K^b^ restricted CD8^+^ T cell epitope SIINFEKL was swapped with D^d^ restricted viral CD8^+^ T cell epitope of m164 _167_AGPPRYSRI_175_. **(B)** Multi-step growth kinetics assay on mouse embryonic fibroblasts (MEF) comparing wild-type (WT) MCMV, RAE-1γMCMV, and MULT-1MCMV is shown. **(C,D)** MEFs were infected with 1.5 PFU/cell of indicated viruses and expression of NKG2D ligands was evaluated 24 h after infection by staining either with **(C)** mouse NKG2D-Fc fusion protein (black line) or **(D)** αRAE-1γ (upper row, black line), αMULT-1 (lower row, black line), and appropriate isotype controls (gray). **(E)** SVEC4-10 cells were infected with 3 PFU/cell of WT MCMV, Δ*m145*MCMV, and MULT-1MCMV for 16 h. Surface expression of MULT-1 was detected with αMULT-1 (black line) or isotype control (gray).

MULT-1MCMV was constructed by replacing the *m145* gene, encoding a viral inhibitor of MULT-1, with the gene encoding MULT-1 under the control of hMIEP (Figure 1A). The construction of RAE-1γMCMV was described previously (21, 22). To study CD8^+^ T cell response induced by these recombinant viruses against well characterized CD8^+^ T cell epitope, all viruses used in this study expressed the immunodominant K^b^ epitope SIINFEKL in place of D^d^ restricted antigenic m164 epitope (Figure 1A). MCMV expressing only SIINFEKL was used as a control (hereby referred as WT MCMV) (25). MULT-1MCMV replication *in vitro* was comparable to WT MCMV and RAE-1γMCMV (Figure 1B). To measure the expression of NKG2D ligands on the surface of infected cells, MEF were infected with WT MCMV, Δ*m152*MCMV, RAE-1γMCMV, Δ*m145*MCMV, and MULT-1MCMV for 24 h. To assess the expression of exogenous NKG2D ligands as well as endogenous NKG2D ligands affected by the deletion of viral evasins, staining was performed using mouse NKG2D-Fc fusion protein (Figure 1C). Expression of inserted NKG2D ligands on the surface of RAE-1γMCMV or MULT-1MCMV infected cells was confirmed by staining with specific antibodies against RAE-1γ and MULT-1, respectively (Figure 1D). As previously published, WT MCMV downregulated NKG2D ligands from the cell surface and deletion of *m152* substantially restored RAE-1 surface expression (35). However, infection of MEF with MULT-1MCMV resulted in high surface expression of MULT-1. Furthermore, both RAE-1γMCMV and MULT-1MCMV infected cells showed similar binding of NKG2D-Fc (Figure 1C). Since MEF constitutively express very low level of MULT-1, the effect of *m145* deletion on the expression of this ligand was hardly detectable. However, the impact of m145 on the surface expression of MULT-1 was detectable after infecting SVEC4-10 cells that constitutively express MULT-1 (Figure 1E) (18, 19). Altogether, these data demonstrate that cells infected with MULT-1MCMV express high levels of MULT-1 on their surface and its expression was not abrogated by function of other viral regulator of MULT-1.

### MULT-1MCMV Is Strongly Attenuated in Adult and Neonatal Mice

**Figure 2:**
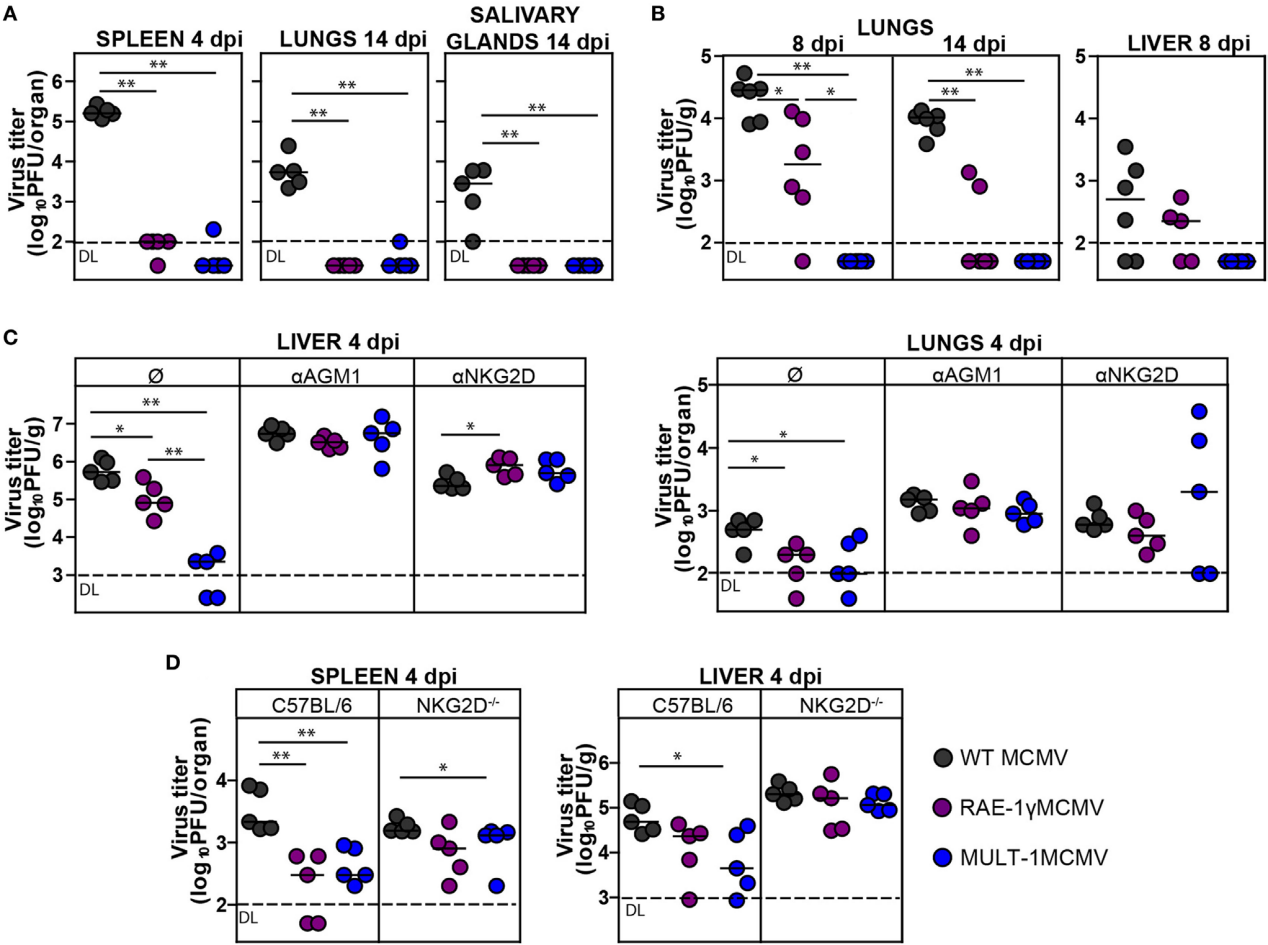
Recombinant murine CMV (MCMV) expressing MULT-1 is controlled by NK cells in NKG2D-dependent manner. **(A)** BALB/c mice were infected intravenously (i.v.) with 2 × 10^5^ PFU of wild-type (WT) MCMV, RAE-1γMCMV, and MULT-1MCMV. On day 4 and 14 after infection viral titer was determined in organs by plaque assay. **(B)** Newborn C57BL/6 mice were infected with 200 PFU of WT MCMV, RAE-1γMCMV, and MULT-1MCMV intraperitoneally (i.p.) 24 h after birth. Viral titer was determined in organs by plaque assay on day 8 and 14 after infection. **(C)** BALB/c mice received 20 µl of αAGM1 or 250 µg of NKG2D blocking antibody i.p. 2 h before infection and additional dose of NKG2D blocking antibody on day 2 after i.v. infection with 2 × 10^5^ PFU of WT MCMV, RAE-1γMCMV, and MULT-1MCMV. Viral titer was determined in organs by plaque assay on day 4 after infection. **(D)** C57BL/6 and NKG2D^−/−^ mice were i.v. infected with 5 × 10^5^ PFU of WT MCMV, RAE-1γMCMV, and MULT-1MCMV. On day 4 after infection viral titer was determined in organs by plaque assay. Each circle represents an individual animal and lines represent medians. Data were analyzed using Mann–Whitney *U* test. Asterisks denote significant values: *P < 0.05; **P < 0.01. Abbreviation: DL, detection limit.

To determine how expression of high affinity NKG2D ligand MULT-1 affects viral control *in vivo*, BALB/c mice were infected with WT MCMV, RAE-1γMCMV, and MULT-1MCMV i.v. and viral titers were analyzed at different times after infection. By the day 4 after infection both RAE-1γMCMV and MULT-1MCMV were heavily attenuated in spleen, lungs, and liver (Figures 2A,C). Moreover, in liver MULT-1MCMV was even more attenuated as compared to RAE-1γMCMV (Figure 2C). Both RAE-1γMCMV and MULT-1MCMV were also attenuated in lungs at day 14 post infection compared to WT MCMV and were completely undetectable in salivary glands (Figure 2A). As shown previously (18), Δ*m145*MCMV was attenuated as compared to WT MCMV, but attenuation of MULT-1MCMV was even stronger (Figure S1A in Supplementary Material).

We have previously shown that RAE-1γMCMV was attenuated in immunologically immature newborns (21). Here, we show that MULT-1MCMV was severely attenuated in newborn mice as well (Figure 2B). Surprisingly, MULT-1MCMV was controlled more rapidly than RAE-1γMCMV in infected newborns, with virus undetectable at the day 8 after infection.

To identify the immune mechanism responsible for the efficient control of MULT-1MCMV, groups of infected BALB/c mice were treated either with αAGM1 to deplete NK cells or NKG2D blocking antibody. Blocking of NKG2D receptor abolished the differences between mutant viruses and WT MCMV suggesting that early attenuation of recombinant viruses expressing NKG2D ligands is exclusively NKG2D-dependent. Depletion of NK cells by αAGM1 also resulted in the abrogation of differences in viral titer between the groups (Figure 2C). It has been well established that NK cells in MCMV resistant C57BL/6 mice control virus *via* a direct recognition of viral m157 protein by Ly49H activating receptor (36). Notably, viruses expressing NKG2D ligands were more sensitive to NK cell-dependent control even in C57BL/6 mice but this phenotype was lost in NKG2D^−/−^ mice (Figure 2D).

### MULT-1MCMV Induces a Strong and Functional Antigen-Specific CD8^+^ T Cell Response

**Figure 3:**
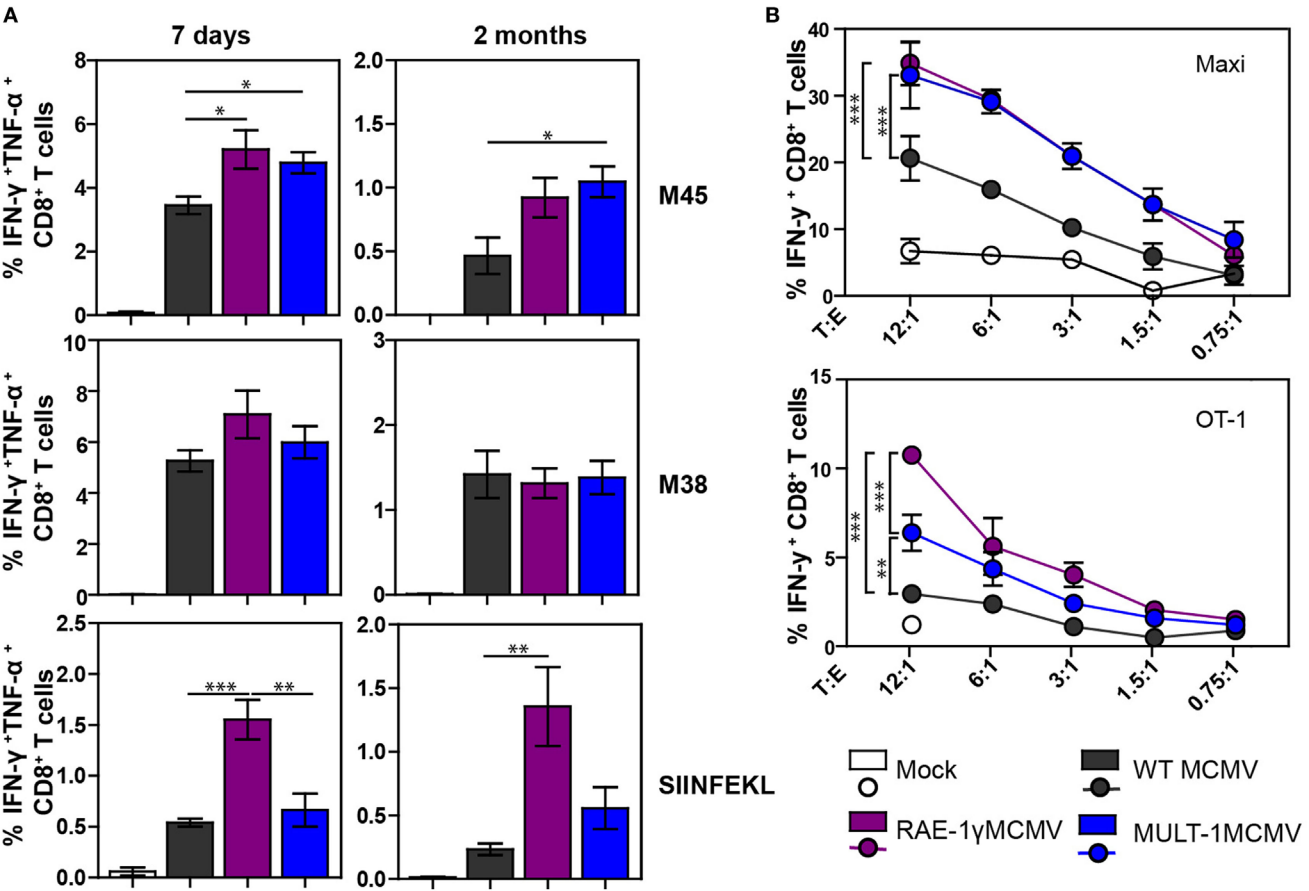
Attenuated MULT-1MCMV induces strong antigen-specific CD8^+^ T cell response and efficiently stimulates CD8^+^ T cells *in vitro*. C57BL/6 mice were infected footpad with 2 × 10^5^ PFU of wild-type (WT) MCMV, RAE-1γMCMV, and MULT-1MCMV. **(A)** At different times after infection CD8^+^ T cells from spleen were analyzed after peptide stimulation for cytokine production (*n* = 5). **(B)** C57BL/6 bone marrow-derived dendritic cells (BMDCs) were infected with 2 PFU/cell of WT MCMV, RAE-1γMCMV, or MULT-1MCMV. After 24 h, infected BMDCs were co-incubated with splenocytes from Maxi or OT-1 mice for 6 h and IFN-γ production was measured by flow cytometry. Representative data from two to five independent experiments are shown. Data are presented as mean ± SEM and were analyzed using Student’s *t*-test. Asterisks denote significant values: *P < 0.05; **P < 0.01; ***P < 0.001.

We have already shown that MCMV expressing NKG2D ligand RAE-1γ induces a strong CD8^+^ T cell response despite its attenuation *in vivo* (21, 22). To examine CD8^+^ T cell response after MULT-1MCMV infection, C57BL/6 mice were infected into footpad with WT MCMV, RAE-1γMCMV, and MULT-1MCMV. Antigen-specific CD8^+^ T cell response was evaluated in the early (7 days) and late (2 months) phase of infection by measuring IFN-γ and TNF-α production after stimulation with non-inflationary peptide M45, inflationary peptide M38, as well as OVA-derived peptide SIINFEKL (Figure 3A; Figure S2 in Supplementary Material). At both time points after infection the frequencies and absolute numbers of virus-specific CD8^+^ T cells were comparable or higher in MULT-1MCMV immunized animals compared to WT MCMV. While virus-specific CD8^+^ T cell response was similar in RAE-1γMCMV and MULT-1MCMV, RAE-1γMCMV induced more CD8^+^ T cells specific for the vectored epitope SIINFEKL. Furthermore, RAE-1γMCMV induced more effector memory SIINFEKL-specific CD8^+^ T cells than MULT-1MCMV, while frequency of M38-specific memory subsets was comparable between viruses (Figure S3 in Supplementary Material). Of note, although MULT-1MCMV was attenuated compared to Δ*m145*MCMV, both viruses induced similar CD8^+^ T cell response (Figure S1B in Supplementary Material).

To circumvent the impact of a different antigenic load, we performed an *in vitro* stimulation of CD8^+^ T cells. BMDCs were infected with WT MCMV, RAE-1γMCMV, and MULT-1MCMV and co-incubated with splenocytes isolated from naive TCR transgenic mice possessing CD8^+^ T cells specific for the M38 epitope (Maxi) or SIINFEKL (OT-1) (Figure 3B). MULT-1MCMV infected BMDCs stimulated a higher proportion of Maxi CD8^+^ T cells to produce IFN-γ compared to WT MCMV and equal to RAE-1γMCMV. In accordance with data presented in Figure 3A, RAE-1γMCMV infected BMDCs provided superior stimulation of OT-1 CD8^+^ T cells. However, MULT-1MCMV infected BMDCs stimulated OT-1 CD8^+^ T cells better than WT MCMV. Altogether, MULT-1MCMV induced a robust antigen-specific CD8^+^ T cells response to both viral and vectored epitopes in both acute and latent phase of infection.

**Figure 4:**
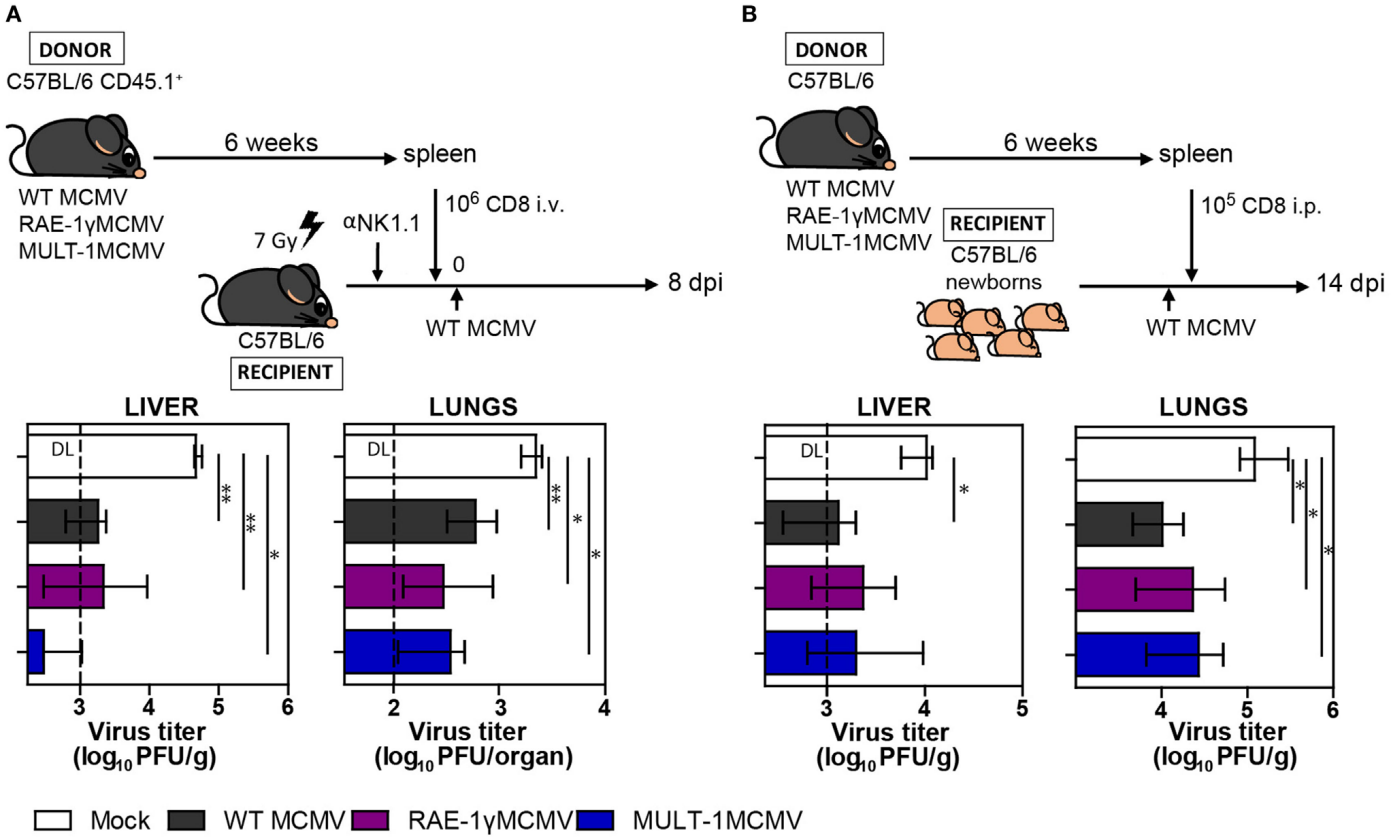
MULT-1MCMV induces protective anti-viral CD8^+^ T cell response. **(A)** C57BL/6 CD45.1^+^ mice were infected footpad (f.p.) with 2 × 10^5^ PFU of wild-type (WT) MCMV, RAE-1γMCMV, and MULT-1MCMV. After 6 weeks 10^6^ CD8^+^ T cells sorted from spleens were intravenously transferred into NK depleted, irradiated C57BL/6 recipients challenged f.p. with 10^5^ PFU of WT MCMV. Eight days after infection organs of recipient animals were collected and viral titer was determined by plaque assay (*n* = 4–8). **(B)** C57BL/6 mice were infected f.p. with 2 × 10^5^ PFU of WT MCMV, RAE-1γMCMV, and MULT-1MCMV and after 6 weeks 10^5^ CD8^+^ T cells sorted from spleen were transferred into C57BL/6 newborn mice challenged i.p. with 500 PFU of WT MCMV. On day 14 after challenge infection viral titer was determined in organs by plaque assay (*n* = 4–6). Representative data of two independent experiments are shown. Data are presented as medians with interquartile range and analyzed using Mann–Whitney *U* test. Asterisks denote significant values: *P < 0.05; **P < 0.01. Abbreviation: DL, detection limit.

Long-term CD8^+^ T cell memory formation is critical for protection upon challenge infection later in life. To assess the protective capacity of virus-specific CD8^+^ T cells induced by MULT-1MCMV, adoptive transfer experiments were performed. CD8^+^ T cells were sorted from C57BL/6 CD45.1^+^ donor mice latently infected with WT MCMV, RAE-1γMCMV, and MULT-1MCMV and adoptively transferred into NK depleted and irradiated C57BL/6 CD45.2^+^ recipients infected with WT MCMV (Figure 4A). CD8^+^ T cells from MULT-1MCMV immunized donors controlled the infection in lungs and liver of challenged recipients more efficiently than non-immune CD8^+^ T cells and equally efficient as CD8^+^ T cells obtained from WT MCMV and RAE-1γMCMV immunization. By using a similar approach, we evaluated the protective capacity of MULT-1MCMV immunization by transferring immune CD8^+^ T cells into newborn mice infected with WT MCMV (Figure 4B). CD8^+^ T cells derived from WT MCMV, RAE-1γMCMV, and MULT-1MCMV infected mice provided a similar level of protection in this model as well. Altogether, our results demonstrated that antigen-specific CD8^+^ T cells induced by highly attenuated MULT-1MCMV are functional and protective against MCMV infection.

### Immunization of Female Mice With MULT-1MCMV Provides Antibody Mediated Protection to Their Offspring

**Figure 5:**
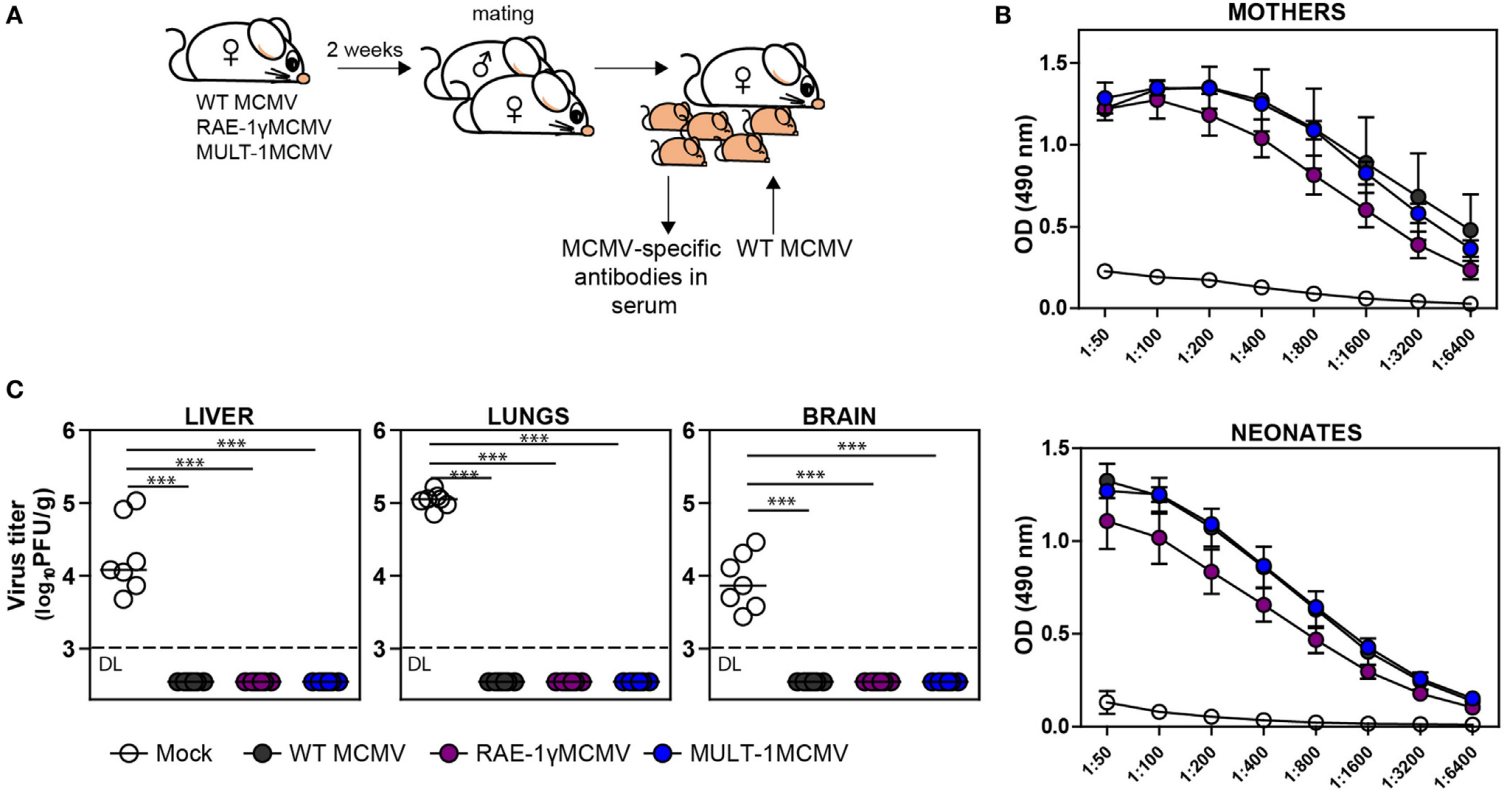
Vaccination of female mice with MULT-1MCMV induces MCMV-specific antibodies which protect their offspring from MCMV challenge. **(A)** BALB/c females were immunized intravenously with 2 × 10^5^ PFU of wild-type (WT) MCMV, RAE-1γMCMV, and MULT-1MCMV 2 weeks prior mating. Six hours after birth, their offspring were either bled to collect sera or intraperitoneally challenged with 500 PFU of WT MCMV. **(B)** Titers of MCMV-specific antibodies in sera of immunized mothers (in average 1.5 month after infection) and their offspring (6 h after birth) are shown. **(C)** WT MCMV challenged newborn mice were sacrificed 9 days after infection and viral titer was determined in their organs by plaque assay. Each circle represents an individual animal. Results are presented as pooled data from two independent experiments. Data are presented as mean ± SEM **(B)** or medians **(C)** and were analyzed using two-way ANOVA **(B)** and Mann–Whitney *U* test **(C)**. Asterisks denote significant values: *P < 0.05; **P < 0.01; ***P < 0.001. Abbreviation: DL, detection limit.

Induction of protective antibody response is a favorable feature of any vaccine. To determine whether MULT-1MCMV can induce MCMV-specific antibodies in immunized mothers which could protect their offspring from MCMV disease, BALB/c females were infected with WT MCMV, RAE-1γMCMV, and MULT-1MCMV 2 weeks prior mating (Figure 5A). Analysis of immune sera showed that MULT-1MCMV immunized mothers, as well as their offspring, had a substantial level of MCMV-specific antibodies (Figure 5B). There was no difference in levels of anti-viral antibodies between MULT-1MCMV compared to WT MCMV. As shown previously, slightly lower antibody titer was observed in mice immunized with RAE-1γMCMV (21). Moreover, newborns from MULT-1MCMV immunized mothers were completely protected upon infection with WT MCMV, as well as newborns from WT MCMV and RAE-1γMCMV immunized dams (Figure 5C). Altogether, these data show that despite a strong attenuation, MULT-1MCMV immunization of mothers induces a strong production of anti-viral antibodies, which can pass the placenta and protect their offspring from MCMV disease.

## Discussion

After resolution of primary CMV infection, CD8^+^ T cells specific for certain immunodominant epitopes are not maintained as a low abundant memory population, but rather gradually increase in frequency acquiring an effector-like phenotype, a phenomenon known as “memory inflation” (37, 38). This characteristic of CMV induced CD8^+^ T cell response could be exploited in the development of CD8^+^ T cell-based live vaccines (39–42). Live attenuated vaccines imitate natural infection by inducing a broad cellular and humoral response to variety of antigens, which makes them superior to subunit vaccines. Still, there are concerns about using live virus as a vaccine as it might cause unwanted virulence, especially in high-risk individuals such as immunocompromised patients. CMV has many nonessential genes involved in subversion of immune response and the deletion of those genes enables the manipulation of its virulence and the quality of immune response. In our previous studies we took advantage of virus lacking the NKG2D immunoevasin to generate recombinant immunologically attenuated vaccine vector RAE-1γMCMV that retained the ability to induce a strong adaptive response (21–23). NKG2D ligands are differently expressed and regulated in tissues, they differ in the affinity for the receptor and certain ligands are restricted to the particular mouse strains (11, 43–45). MULT-1 has the highest affinity for the receptor among all mouse NKG2D ligands and its transcripts were found in most of the healthy tissues, but its protein expression was strictly regulated on posttranslational level (46). To exploit those differences in the context of a MCMV-based vaccine, we constructed MULT-1MCMV and compared it with WT MCMV and RAE-1γMCMV. MULT-1MCMV was highly immunologically attenuated, but nevertheless induced a strong and protective antigen-specific CD8^+^ T cell and antibody response. Thus, this study further supports the idea that attenuated recombinant CMVs expressing NKG2D ligands can be used as efficient vaccines and vaccine vectors.

It is well known that NK cell response can also shape the adaptive immune response (47). The most extensively studied example of strong NK control of MCMV infection is the direct recognition of viral protein m157 by the activating NK receptor Ly49H, leading to efficient virus control (36, 48). In most cases, a strong NK control would impair CD8^+^ T cell responses presumably due to the lower viral burden and cytokine milieu diminishing antigen presentation (49, 50). Indeed, during MCMV infection the lower viral burden reduces the magnitude of long-term CD8^+^ T cell response but does not impact its kinetics (51, 52). Paradoxically, it has been shown that blocking viral replication increases CD8^+^ T cell response to non-inflationary epitopes presented by preserved dendritic cells (DCs) which would otherwise be depleted due to type I IFNs (53, 54). Interestingly, this effect was not evident in the population of inflationary CD8^+^ T cells. While convectional non-inflationary CD8^+^ T cells are preferentially primed by cross-presented antigens on DCs (55), inflationary CD8^+^ T cells are originating from cells primed early in infection as well as from constantly replenished short-lived effectors (56) maintained at high levels by sporadic virus reactivations in non-hematopoietic cells (30, 57). Differential requirements in generation of non-inflationary and inflationary CD8^+^ T cells in MCMV infection might explain these findings (58). Here we show that despite efficient NK-mediated virus control, MULT-1MCMV induces CD8^+^ T cell response of similar or greater magnitude compared to WT MCMV to both non-inflationary and inflationary epitopes. While all viruses induced comparable CD8^+^ T cell response to viral antigens, RAE-1γMCMV induced a higher number of SIINFEKL-specific CD8^+^ T cells. Somewhat better response to non-inflationary epitope M45 in mice infected with recombinant viruses expressing NKG2D ligands could be explained either by a better cross-presentation due to preserved DCs in absence of strong infection or immune function of these proteins (21, 22, 54). The strong engagement of NKG2D and activation of NK cells might result in an environment favoring antigen presentation. Alternatively, the co-stimulation signal by engagement of NKG2D on CD8^+^ T cells could rescue T cell responses which would otherwise be weaker due to strong viral control and action of other viral evasins. The importance of CD8^+^ T cell co-stimulation is evident from the fact that TCR signaling without an appropriate co-stimulation signal drives CD8^+^ T cells to the state of anergy rather than activation (59). CMVs have developed multiple mechanisms to evade CD8^+^ T cell recognition of infected cells. Indeed, MCMV possess several genes encoding proteins which interfere with antigen presentation by downregulating the expression of MHC I molecules (6). Ligands for the major T cell co-stimulatory receptor CD28 are also targeted by viral immunoevasion (59–61). CD28/B7-mediated co-stimulation is indispensable in acute infection and for the establishment of CD8^+^ T cell memory in MCMV (62). In LCMV infection, the absence of B7-mediated co-stimulation can be substituted with other co-stimulatory pathways (63). Though this was not shown for CD8^+^ T cell response in herpes viruses, it suggests that under certain conditions co-stimulatory pathways might act compensatory to each other. Our *in vitro* studies showed that RAE-1γMCMV and MULT-1MCMV infected BMDCs have an improved capacity to stimulate CD8^+^ T cells compared to WT MCMV. The engagement of co-stimulatory receptor NKG2D on CD8^+^ T cells was reported to promote proliferation and cytotoxic capacity of antigen-specific CD8^+^ T cells in various experimental settings (64–66), as well as rescue memory of unhelped CD8^+^ T cells (67). Moreover, it was shown recently that engagement of NKG2D on NK cells augments their expansion during MCMV infection (68). Therefore, we hypothesize that co-stimulatory function of NKG2D on CD8^+^ T cells might overcome multiple ways of viral interference with antigen presentation and downregulation of co-stimulatory molecules resulting in a better stimulation and improved CD8^+^ T cell responses *in vivo*.

Apart from prompting durable CD8^+^ T cell memory, the ability to induce humoral response is another vital feature of CMV vaccine, since antigen-specific antibodies are able to cross the placental barrier and are the first to protect the fetus and newborns against congenital infection (69, 70). Despite the lower antigenic load, MULT-1MCMV immunization induced MCMV-specific antibodies capable of protecting offspring against a challenge infection.

Another interesting finding of this study was the rapid attenuation of MULT-1MCMV pronounced in perinatally infected newborns. This is in agreement with our recent study showing that MULT-1MCMV failed to reach the brain of mice infected as newborns resulting in the absence of brain inflammation and establishment of tissue-resident memory CD8^+^ T cells (71). NK cells are immature in newborns, at least partially due to the presence of high levels of immune suppressive TGF-β, which makes newborns highly susceptible to viral infections (72). Since NKG2D is expressed early in ontogeny of NK cells (73), we speculate that the engagement of NKG2D with a high affinity ligand might overcome the suppressive environment in newborn mice, but the exact mechanism of MULT-1MCMV control in newborns remains elusive. Yet, this study demonstrates the feasibility of CMV-based vaccine vectors to be used even in newborns.

Altogether, we show for the first time that MCMV expressing the high affinity NKG2D ligand MULT-1 has numerous favorable features of a vaccine such as being highly attenuated, but still able to induce both cellular and humoral adaptive immunity. We have established the vaccine properties of MULT-1MCMV against MCMV infection, which does not exclude the prospect to test its capacity as a vector vaccine. In conclusion, by comparing recombinant viruses expressing different NKG2D ligands, we confirmed the dual role of NKG2D in mediating a strong virus control, while retaining CD8^+^ T cell response equivalent or better than WT virus infection, which can serve as a model approach for the development of a similar HCMV-based vaccine vector.

## Ethics Statement

This study was carried out in accordance with the recommendations of Regulations on the protection of animals used for scientific purposes (Official Gazette of the Republic of Croatia, 55/2013). Ethics Committee of the Veterinary Department of the Ministry of Agriculture and Animal Welfare Committee of the University of Rijeka Faculty of Medicine approved all animal experiments.

## Author Contributions

LH and IB designed the study, performed the experiments, analyzed the data, and wrote the manuscript. TJ and JR designed and generated recombinant viruses. VL and SJu contributed to performing experiments and critical reading of the manuscript. AK and SJo designed and oversaw the study and wrote the manuscript.

## Conflict of Interest Statement

The authors declare that the research was conducted in the absence of any commercial or financial relationships that could be construed as a potential conflict of interest.
