# Supplementary material for: Murine CMV Expressing the High Affinity NKG2D Ligand MULT-1: A Model for the Development of Cytomegalovirus-Based Vaccines
Source: Front Immunol. 2018 May 7;9:991. doi: 10.3389/fimmu.2018.00991 (PMC5949336; doi:10.3389/fimmu.2018.00991)
Supplement: Supplementary file 1 [file Presentation_1.PDF]

Supplementary Material

MCMV expressing the high affinity NKG2D ligand MULT-1: a model for the development of CMV based vaccines

Lea Hiršl[1], Ilija Brizić [1], Tina Jenuš[1], Vanda Juranić Lisnić[1,2], Johanna Julia Reichel[1], Slaven Jurković[3], Astrid Krmpotić[2], Stipan Jonjić[1,2*]

[1] Center for Proteomics, University of Rijeka, Faculty of Medicine, Rijeka, Croatia

[2] Department of Histology and Embryology, University of Rijeka, Faculty of Medicine, Rijeka, Croatia

[3] Medical Physics Department, University Hospital Rijeka, Rijeka, Croatia; Department of Physics, University of Rijeka, Faculty of Medicine, Rijeka, Croatia

*** Correspondence:**
Stipan Jonjic, stipan.jonjic@medri.uniri.hr

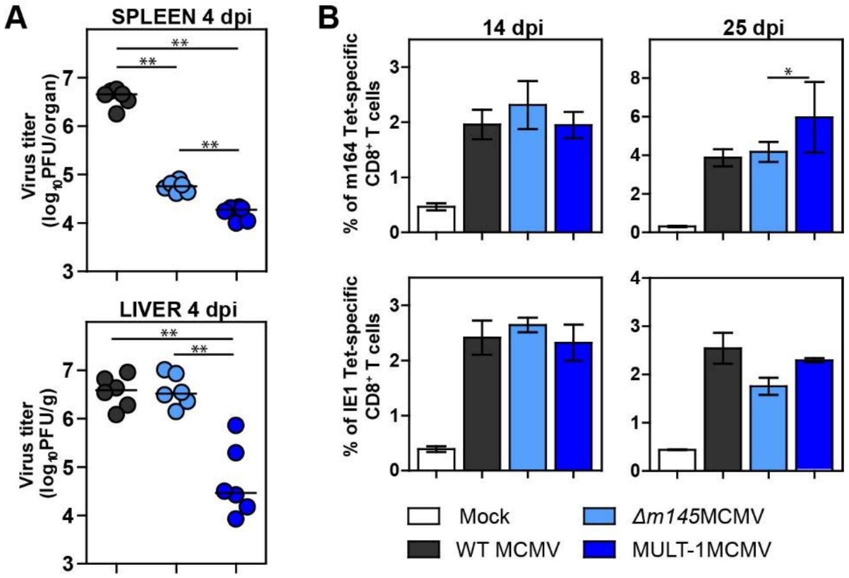

Supplementary Figure 1. MULT-1MCMV is more attenuated than *Δm145*MCMV but induces comparable CD8^{+} T cell response. (A) BALB/c mice were infected i.v. with 2 x 10^5 PFU of WT MCMV, *Δm145*MCMV and MULT-1MCMV (without SIINFEKL). On day 4 after infection organs were collected and viral load was determined by plaque assay. **(B)** BALB/c mice were infected f.p. with 2 x 10^5 PFU of WT MCMV, *Δm145*MCMV and MULT-1MCMV. At different time points after infection frequency of m164 and IE1 tetramer-specific CD8^{+} T cells was determined in spleen. Data are presented as median **(A)** or means ± SEM **(B)** and were analyzed using Mann-Whitney U test **(A)** or Student's t-test **(B)**. Asterisks denote significant values: *P < 0.05; **P < 0.01.

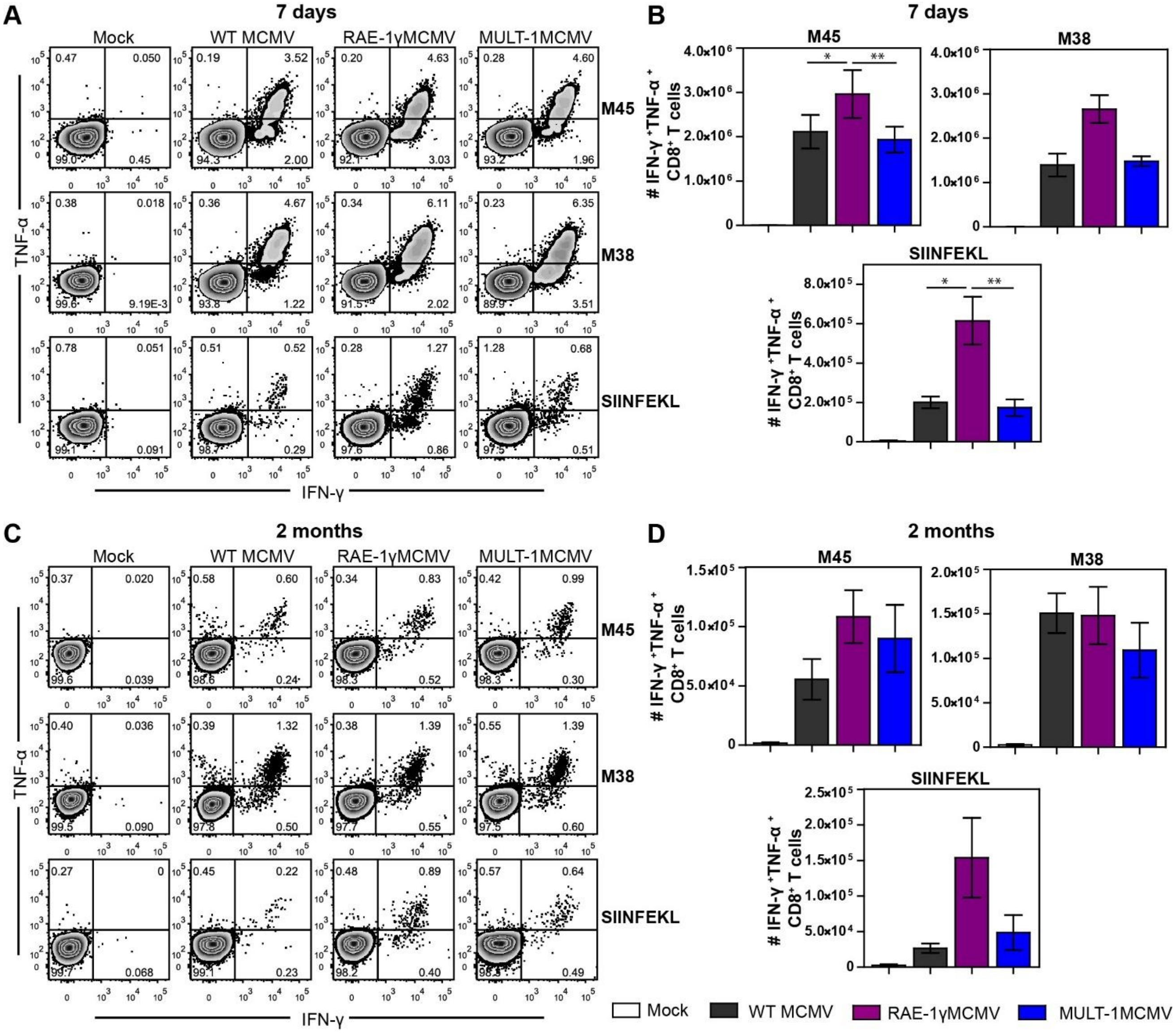

Supplementary Figure 2. Attenuated MULT-1MCMV induces strong antigen-specific CD8⁺ T cell response. C57BL/6 mice were infected f.p. with 2 x 10^5 PFU of WT MCMV, RAE-1γMCMV and MULT-1MCMV. At different times after infection $CD8^+$ T cells from spleen were analyzed after peptide stimulation. **(A, C)** Representative flow cytometry data showing IFN-γ and TNF-α production gated on live $CD8^+$ T cells. **(B, D)** Absolute number of antigen-specific cytokine producing $CD8^+$ T cells in spleens of immunized animals. Data are presented as means ± SEM **(B, D)** and were analyzed using Student's t-test **(B, D)**.

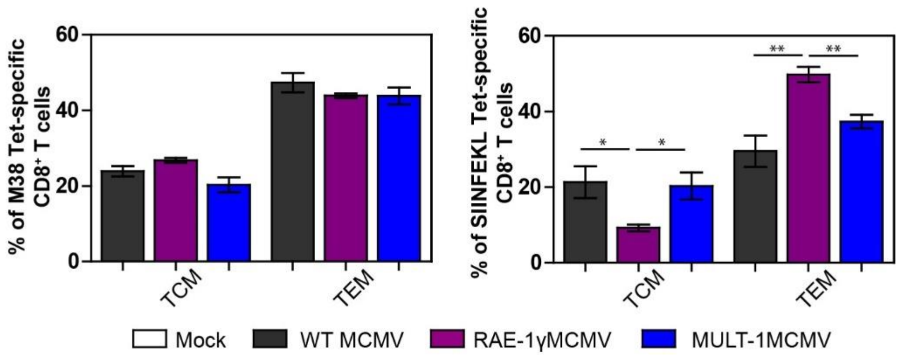

Supplementary Figure 3. Attenuated MULT-1MCMV induces CD8$^+$ T cell memory subsets comparable to WT MCMV. C57BL/6 mice were infected f.p. with 2 x 10^5 PFU of WT MCMV, RAE-1γMCMV and MULT-1MCMV. Four months after infection tetramer-specific CD8$^+$ T cells from spleens of immunized animals were analyzed for memory CD8$^+$ T cell phenotype as central memory CD44^{hi}CD127^{hi}CD62L^{hi} (TCM) and effector memory CD44^{hi}CD127^{hi}CD62L^{neg} (TEM). Data are presented as means ± SEM and were analyzed using Student's t-test. Asterisks denote significant values: *$P < 0.05$; **$P < 0.01$.
